# Autosomal Recessive Cerebellar Ataxias: Translating Genes to Therapies

**DOI:** 10.1002/ana.27271

**Published:** 2025-06-04

**Authors:** Brent L. Fogel, Thomas Klopstock, David R. Lynch, Francesca Maltecca, Mayank Verma, Berge A. Minassian, Frances M. Platt, Débora Farina Gonçalves, Hélène Puccio, Andreas Roos, Matthis Synofzik

**Affiliations:** ^1^ Departments of Neurology and Human Genetics, David Geffen School of Medicine University of California Los Angeles Los Angeles CA; ^2^ Department of Neurology with Friedrich‐Baur‐Institute University Hospital of Ludwig‐Maximilians‐Universität München Munich Germany; ^3^ German Center for Neurodegenerative Diseases (DZNE) Munich Germany; ^4^ Munich Cluster for Systems Neurology (SyNergy) Munich Germany; ^5^ Division of Neurology Children's Hospital of Philadelphia Philadelphia PA; ^6^ Mitochondrial Dysfunctions in Neurodegeneration Unit, Division of Neuroscience IRCCS Ospedale San Raffaele Milan Italy; ^7^ Division of Neurology, Department of Pediatrics University of Texas Southwestern Medical Center Dallas TX; ^8^ Department of Pharmacology University of Oxford Oxford UK; ^9^ Institut NeuroMyoGène (INMG), Unité Physiopathologie et Génétique du Neurone et du Muscle Université Claude Bernard Lyon 1 CNRS UMR 5261, Inserm U1315 Lyon France; ^10^ Department of Pediatric Neurology, Centre for Neuromuscular Disorders, Centre for Translational Neuro‐ and Behavioral Sciences University Duisburg‐Essen Essen Germany; ^11^ Brain and Mind Research Institute, Children's Hospital of Eastern Ontario Research Institute Ottawa Canada; ^12^ Department of Neurology, Medical Faculty and University Hospital Düsseldorf Heinrich Heine University Düsseldorf Germany; ^13^ Division Translational Genomics of Neurodegenerative Diseases, Hertie‐Institute for Clinical Brain Research and Center of Neurology University of Tübingen Tübingen Germany; ^14^ German Center for Neurodegenerative Diseases (DZNE) Tübingen Germany

## Abstract

Autosomal recessive cerebellar ataxias (ARCAs) represent over 200 clinically heterogeneous genetic conditions involving degeneration of the cerebellum and associated tracts with resultant impairment of balance and coordination. Advancements in genomic testing have enabled rapid identification of the majority of known recessive disorders, shifting research focus to the development of targeted mechanistic treatments addressing underlying physiological pathways. Molecular classification allows recognition of cellular, biochemical, and genetic targets for high‐effect precision therapy development. ARCAs represent a significant global health burden, requiring establishment of a robust pathway for novel therapeutic discovery through modification of mechanisms of disease pathogenesis and subsequent clinical trial development. ANN NEUROL 2025;98:448–470

## Graphical abstract

Autosomal recessive cerebellar ataxias are disabling neurodegenerative genetic conditions affecting balance and coordination. Advancements in genomic testing have improved diagnosis, leading to a new focus on the development of targeted precision therapeutics addressing cellular, biochemical, and genetic disease mechanisms with a resulting emphasis on the development of new clinical trial strategies. Image created in BioRender.

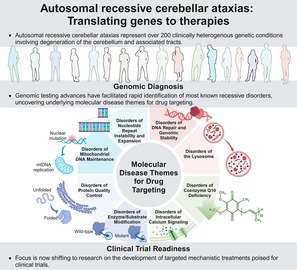

Autosomal recessive cerebellar ataxias (ARCAs) represent a diverse category of over 200 clinically heterogeneous genetic conditions that lead to degeneration of the cerebellum and/or its associated afferent tracts (eg, spinocerebellar or dorsal columns).^1^, ^2^, ^3^ They are often associated with progressive damage to additional extra‐cerebellar neurological systems, in particular the corticospinal tracts (resulting in >100 spastic ataxias, substantially overlapping with hereditary spastic paraplegias),^4^, ^5^, ^6^ and dorsal root ganglia/peripheral nerves (causing polyneuropathy),^7^, ^8^ but also the cerebral cortex, basal ganglia, and/or retina.^3^

Genomic testing, specifically whole exome sequencing (WES) and whole genome sequencing (WGS), has proved extremely effective at achieving a diagnosis in between one‐quarter to one‐half of all patients across most studies,^4^, ^6^, ^9^, ^10^ and likely even more with incorporation of new bioinformatic tools that predict copy number and short tandem repeat variants in both coding and non‐coding regions.^9^ Tools such as ExpansionHunter,^11^ and ExpansionHunter DeNovo^12^ have yielded recent discoveries of high prevalence novel disorders,^13^, ^14^, ^15^ and broad detection of repeat expansions from WGS.^16^ As nucleotide repeat expansions remain the most common pathogenic mechanism in ARCAs, as in dominant ataxias, WGS allows their detection, including the two most common (Friedreich ataxia [FRDA] and *replication factor c subunit 1* [*RFC1*]‐mediated ataxia).^10^, ^17^, ^18^ These genetic advances have now revolutionized the treatment perspective, previously just symptomatic management, by promising therapies targeting the underlying molecular pathologic mechanisms.^19^, ^20^

## Autosomal Recessive Cerebellar Ataxias

The cerebellum plays a critical role in the integration of sensory input and motor output to generate smooth and coordinated movement. Damage to the cerebellum leads to cerebellar ataxia, impairment of balance and coordination despite normal muscular strength.^1^, ^2^, ^3^, ^21^ An extensive and diverse array of causes can lead to this,^22^ however, approximately half^23^ are because of Mendelian genetic etiologies stemming from an ever‐expanding catalog of causative mutations involving nearly 600 genes and 1,000 phenotypes, with over 200 of those genes resulting in more than 600 phenotypes associated with autosomal recessive inheritance (see Supplemental Material).^3^, ^24^ As this number has grown, individual recessive disorders were initially categorized under the heading of ARCAs. More recently, the term spinocerebellar ataxias, recessive (SCARs) has partly been used, primarily in the diagnostic laboratory setting, however, in clinical practice this can lead to confusion with the dominant cerebellar ataxias commonly designated as the spinocerebellar ataxias (SCAs).^24^ Ultimately all these nomenclature systems have their respective drawbacks,^5^ and direct use of the gene name (rather than a separate numbering system) appears to be the most informative and least confusing approach, as we adopt here. Although individually these disorders are rare, genetic cerebellar ataxia as a whole is not, with an estimated 6 to 13 per 100,000 persons affected worldwide, both adults and children,^23^ and up to 1.0 million persons affected globally, a number that is likely an underestimate because of incomplete sampling across all populations. Recessive ataxias likely represent at least half of these conditions,^23^ however, this estimate is also uncertain.

Clinically, many of the recessive ataxias show a high degree of overlap in terms of clinical presentation and underlying neurological systems, thereby rendering neurological examination findings commonly insufficient to achieve diagnosis without genetic testing. Although ARCAs mostly start before age 40 years,^10^, ^21^ late‐onset phenotypes are increasingly described as spectrum phenotypes of typically early‐onset ARCAs (eg, late‐onset FRDA^18^) or as the onset age of some newly identified ARCAs (eg, *RFC1* ^10^, ^17^, ^25^). Many disorders also involve organ systems outside the central nervous system.^1^, ^2^, ^3^, ^21^ As ARCAs are both chronic and progressive, ultimately resulting in impairments of speech, swallowing, and voluntary movements as well as wheelchair‐dependence and a lack of functional independence because of the inability to perform self‐care,^22^ a significant burden of disease globally will result unless effective treatments can be identified.

## Molecular Disease Themes for Potential ARCA Drug Targets

A focus on molecular mechanisms as drug targets with potentially high‐effect size opens a new perspective for translational ARCA research and will allow prioritization of research and treatment development on molecular pathways—in particular, on molecular themes that might even be shared across several ARCAs. Clinical neurology has seen a rapid burst in the development of molecular therapies targeting underlying genes or genetic mutations responsible for specific disorders with clinically effective United States Food and Drug Administration (FDA)‐approved therapies already available that use antisense oligonucleotides, small interfering RNA, or gene replacement strategies. Targeted disease‐modifying therapies for ARCAs follow a mechanistically inspired approach based on underlying gene, molecular mechanisms, and pathways.^3^ Here, we focus on molecular disease themes in ARCAs that (1) recur in a number of distinct diseases (ie, ≥2 ARCAs genotypes), and (2) where key ARCAs of each theme have potential molecular treatments that follow a mechanistically sound approach, are at least in pre‐clinical stages, and/or would impact a large number of patients. Although these criteria encompass the most clinically relevant disorders, not all well‐established ARCAs (eg, *PNPLA6*, *POLR3A*, *SYNE1*, *SPTBN2*, *TWNK*, *VLDLR*, etc.) could be included, however, these are covered in detail elsewhere.^1^, ^2^, ^3^, ^21^

## Disorders of Nucleotide Repeat Instability and Expansion

### Friedreich Ataxia

The two most common ARCAs are Friedreich Ataxia (FRDA), resulting from an expanded intronic GAA repeat on both alleles of the *FXN* gene, and *RFC1*‐mediated ataxia, due to biallelic intronic AAGGG repeats.^10^ A more complete understanding of these repeat disorders would, therefore, facilitate therapy for a large number of patients.

The discovery of the expanded GAA repeats in *FXN* and the characterization of its role in the FRDA disease process created a pathophysiological explanation that has become more refined over time.^26^, ^27^ The expanded GAA repeat in the *FXN* gene leads to epigenetic silencing of the gene, with the size of the repeat correlating inversely with the level of *FXN* mRNA production.^26^ This, in turn, leads to decreased expression of the gene product frataxin, a small mitochondrial protein involved in iron‐sulfur cluster synthesis. The silencing of the *FXN* gene is incomplete at the tissue level and some residual *FXN* mRNA synthesis and protein synthesis remain (<20% of normal and frequently <5% of normal in model systems). Approximately 2% of patients carry point mutations (in association with a GAA expansion on the opposite allele) that lead to no frataxin production,^27^ but complete frataxin knockout is lethal to almost all cells. Therefore, the features of FRDA reflect extreme frataxin knockdown, not knockout. This shows that FRDA is caused by frataxin protein deficiency, not mRNA deficiency, and defines the therapeutic approach to the disease.

There are two approaches to the treatment of FRDA: modulating downstream effects of frataxin deficiency (mitochondrial dysfunction) or restoration of frataxin. The former is easier through small molecule approaches and has reached some success with the FDA and the European Medicine Agency approval of omaveloxolone, but the response is incomplete (slowing disease progression only minorly by 2.4 points on the mFARS scale) and likely limited in its endurance.^28^, ^29^ This may reflect the diversity of the types of mitochondrial dysfunction, their limited targetability, and the fact that other functions of frataxin (outside of iron–sulfur cluster synthesis and mitochondrial dysfunction) may exist. The second approach involves the restoration of frataxin normal levels. This can occur using direct protein replacement, gene therapy to add a new episomal *FXN* gene, gene editing to repair the mutated gene, or epigenetic therapy to reactivate the silenced alleles.^30^, ^31^, ^32^, ^33^, ^34^, ^35^, ^36^, ^37^, ^38^, ^39^, ^40^ All of these approaches show benefits in cellular and animal systems. Translation to human therapy has proven difficult (Table 1) as the problematic aspect of frataxin restoration is the wide tissue distribution needed to provide complete benefit.^41^ Furthermore, frataxin levels would need to increase in approximately 50% of cells in any tissue, although the actual increase needed per cell may be small (based on mouse models).^42^ Protein replacement may have limited distribution, and present gene therapy vectors transduce the CNS in a limited manner. However, the recent and future development of blood–brain barrier penetrant AAV serotypes might overcome many of these issues, but frataxin toxicity remains an issue if restored levels become too high.^43^ Small molecules (SynTEF, targeting the epigenetic silencing by the GAA repeat itself^36^; or HDAC inhibitors, targeting the epigenetic changes that help mediate silencing^44^) can enter the CNS well and reactivate frataxin expression, but have shown adverse effects in early studies. Future therapeutic advances in FRDA will require improved knowledge of disease pathophysiology and its modification: all functions of frataxin, vector targeting, refinement of small molecules for epigenetic activation, and discovery of novel *FXN* activation approaches.

**TABLE 1:** Active Clinical Trial Approaches in Friedreich Ataxia

General mechanism	Drug	Specific mechanism	Status	Comment reference
Mitochondrial enhancement	Omaveloxolone	NRF2 activator	Approved	Available for patients older than 15; pediatric studies to begin^28^, ^29^
	Vatiquinone	15‐Lipoperoxidase inhibitor/ferroptosis inhibitor/enhancer of oxidative phosphorylation	Finished phase 3	Results under discussion^37^
	Nicotinamide riboside	Enhancement of sirtuins	Phase 2	Recruiting^157^
	MIB‐626	Enhancement of sirtuins	Phase 1/2	Data pending^157^
	Elamipretide	Enhancement of cardiolipin levels	Phase 2	Recruited^39^
	Leriglitazone	PPAR γ activator	Phase 2	Results available^40^
	IMF/DMF	NRF2 activator	Phase 2	Not yet recruiting^38^
	Artesunate	Control of iron deposition	Phase 2	Recruiting^157^
Frataxin restoration	CT1601/Tat‐frataxin	Frataxin protein replacement	Phase 2	Enrolled^30^
	Etravirine	Unknown	Phase 2	Recruiting^157^
	DT216/Gene tac	Epigenetic activation	Preclinical Phase 1	Adverse event in phase 1^36^
	Directed oligonucleotide	Epigenetic activation	Preclinical	Preclinical^34^
	Nicotinamide high dose	Epigenetic activation	Phase 2	Not yet recruiting.^31^
	Cardiac gene therapy (LX2006)	Gene replacement	Phase 1	Two companies^32^
	CNS gene therapy	Gene replacement	Preclinical	Multiple companies^33^, ^35^

### *RFC1* ‐Mediated Ataxia

Similar to FRDA, recent studies have identified another recessive intronic (AAGGG) repeat expansion in *RFC1*, initially associated with the phenotype of cerebellar ataxia, neuropathy, and vestibular areflexia syndrome (CANVAS).^13^, ^25^ Further studies have supported this mutation as a common cause of late‐onset ataxia, and perhaps the second most common recessive ataxia, and expanded the multi‐systemic phenotype to include spinocerebellar ataxia in isolation or in combination with either sensory neuropathy, vestibular dysfunction, autonomic impairment, motor neuron dysfunction, and chronic cough as well as isolated sensory neuropathy.^17^, ^45^, ^46^, ^47^, ^48^ Although the underlying pathogenesis remains unknown for most patients, the finding of patients with nonsense and truncating *RFC1* mutations has implicated a loss‐of‐function mechanism.^49^, ^50^ At present, no effective treatments for *RFC1*‐mediated ataxia exist, however, strategies using gene replacement and gene editing^51^ are in the pre‐clinical stages.

## Disorders of DNA Repair and Genomic Stability

### Ataxia‐Telangiectasia

**FIGURE 1:**
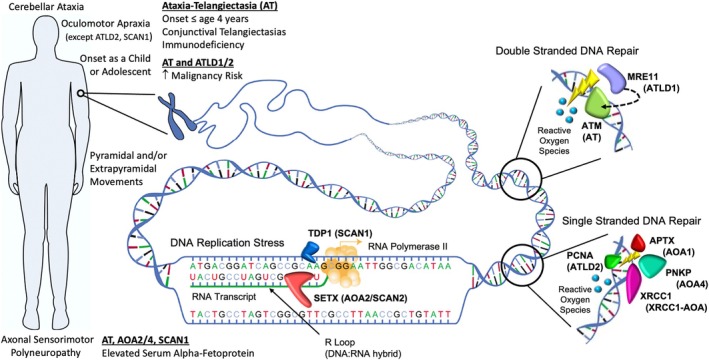
Cerebellar ataxia‐associated proteins involved in DNA repair and genomic stability. Clinical disease features are shown on the left while cellular DNA undergoing transcription is shown on the right with the key functional roles of the disease‐associated proteins indicated. In the presence of double stranded DNA breaks, the ATM protein is activated by a protein complex that includes MRE11. ATM can also be independently activated by reactive oxygen species. Once activated, ATM acts to phosphorylate additional protein factors to initiate DNA repair. In the repair of single strand DNA breaks by excision repair, XRCC1 is recruited to complete the repair and ligate the DNA, a process that can involve PCNA to aid complex formation and function for longer repairs, such as those caused by reactive oxygen species. Additional proteins involved in the process include PNKP, which resolves damage that prevents processing at the end of strand breaks, and APTX, which resolves errors resulting from the DNA ligation process. During transcription, as well as during DNA repair and replication, TDP1 prevents errors that arise during the unwinding of DNA by topisomerase, a process that introduces single strand nicks and therefore also involves XRCC1 and PNKP to repair. SETX, a DNA/RNA helicase, resolves DNA:RNA hybrid R‐loops that form during transcription to maintain genomic stability. Associated ataxic disorders are indicated next to each protein. Please note that it is likely that other cellular functions, aside from DNA repair and genomic stability, including neuronal‐specific functions, likely contribute to disease pathogenesis for many, if not all these proteins. AOA, ataxia with oculomotor apraxia; AT, ataxia‐telangiectasia, ATLD; AT‐like disorder; SCAN, spinocerebellar ataxia with axonal neuropathy. [Color figure can be viewed at www.annalsofneurology.org]

The most common and best studied disorder of DNA repair and genomic stability is ataxia‐telangiectasia (AT), caused by mutation of the *ATM* gene and likely the third most common recessive ataxia in most regions.^52^ Damage to DNA, specifically double stranded DNA breaks, initiates a molecular cascade that triggers activation of the ATM protein (Fig 1).^53^ ATM is a serine/threonine kinase that subsequently phosphorylates hundreds of proteins involved in cell‐cycle control, DNA repair, apoptosis, suppression of chromatin rearrangements, and maintenance of genome stability in response to DNA replication stress.^53^ In addition to its role in DNA repair and related pathways, multiple lines of evidence suggest that, within neurons, ATM also participates in other functions including synaptic vesicle dynamics, calcium homeostasis, autophagy, neurotransmission, and oxidative stress that likely contribute to disease pathogenesis, although the precise mechanisms still remain unclear.^54^, ^55^ Additional disorders in this class are highlighted in Figure 1 and include the AT‐like disorders and the ataxias with oculomotor apraxia (AOAs), such as AOA1 and AOA2.^56^, ^57^, ^58^ Loss‐of‐function mutations in *ATM* lead to the classic form of AT that presents clinically with progressive ataxia often starting before age 4 years as wells as the hallmark conjunctival telangiectasias, oculomotor apraxia, extrapyramidal movements (eg, chorea, dystonia, myoclonus, or tremor), elevated serum α‐fetoprotein (often >1,000μg/L), immunodeficiency with recurrent infections, and an increased risk of developing malignancy, often leukemia or lymphoma.^52^ Less severe *ATM* mutations (eg, leaky splice site or otherwise hypomorphic missense mutations) allow residual ATM kinase activity and give rise to a milder, often later onset, “variant AT” phenotypes, often with predominating dystonia and/or neuropathy phenotypes.^52^

Treatment developments in AT have focused primarily on efforts to restore protein function through molecular interventions. In one example, loss of ATM has been observed to cause downstream hyperactivation of poly‐ADP‐ribose polymerase 1, likely triggering mitochondrial dysfunction and resultant neuronal cell death, which could potentially be countered by nicotinamide supplementation, which has shown benefit in patient trials.^59^ Similarly, dexamethasone administration has shown promise in phase II and III clinical trials.^60^, ^61^ Although the precise mechanism of action is unclear, dexamethasone has been proposed to promote non‐canonical splicing of the endogenous *ATM* mRNA resulting in smaller, but functional variant proteins,^60^ although this remains controversial and more general immunomodulary modes of action should also be considered.^62^ A recent phase III trial of an erythrocyte‐loaded formulation of dexamethasone in 164 children with AT (ATTeST trial) did not meet its primary endpoint, however, a subgroup analysis showed that AT children age 6 to 9 years (still before the most dramatic non‐linear neurological deterioration in natural disease progression) who received high‐dose dexamethasone showed a positive treatment effect on the International Cooperative Ataxia Rating Scale (ICARS) score compared to placebo.^61^ This has led to initiation of a phase III trial focusing specifically on this age group (NEAT trial, NCT06193200).

Still other work has focused on restoring normal ATM expression, particularly through customized splice modulation antisense oligonucleotides (ASOs) that precision target aberrant splice site mutations in individual patients, which might account for up to 5 to 10% of loss‐of‐function mutations in *ATM*,^63^ to restore the normal protein. This includes two recent individualized ASO therapy trials performed in single patients.^63^, ^64^ If successful, this approach might be applicable to a larger range of ARCA conditions, particularly ultra‐rare ARCAs or private mutations where no treatment strategies would otherwise be possible.^65^

### The Ataxias with Oculomotor Apraxia

Similar in clinical presentation to AT, but typically with older onset, are the disorders of AOA, types 1, 2, 4, and *XRCC1*‐AOA. The most common of these is AOA2 (also known as spinocerebellar ataxia with axonal neuropathy type 2 [SCAN2]) caused by mutation in senataxin (*SETX*), an RNA/DNA helicase involved in maintenance of genome integrity in response to DNA replication stress from DNA:RNA hybrid R‐loop formation during transcription, chromosomal stability, DNA damage, autophagy, transcriptional regulation, RNA processing and degradation, and the innate immune response (see Fig 1).^56^, ^57^, ^58^ Loss‐of‐function mutations in *SETX* are associated with an adolescent onset cerebellar ataxia and axonal sensory neuropathy, oculomotor apraxia in more than half of patients, pyramidal and/or extrapyramidal signs (eg, chorea, dystonia, or myoclonus), elevated serum cholesterol, elevated α‐fetoprotein (at least 10‐fold less than in AT), and reduced fertility in males.^66^, ^67^

AOA1, caused by mutation of aprataxin (*APTX*), a gene involved in resolving abortive ligation events that occur during DNA repair (see Fig 1),^68^ typically presents under the age of 10 and shows elevated levels of cholesterol, low serum albumin, and normal α‐fetoprotein levels, in addition to approximately 50% of patients having intellectual disability and a smaller percentage with co‐enzyme Q10 deficiency.^69^ Polynucleotide kinase 3′‐phosphatase (PNKP), a DNA end‐processing protein involved in DNA repair pathways, causes a similar phenotype termed AOA4, likely via cell death because of DNA break accumulation.^69^, ^70^ Mutation of X‐ray cross‐complementing protein 1 (*XRCC1*), a protein scaffolding factor important in base excision repair, causes a similar adult‐onset cerebellar ataxia.^69^, ^71^ Last, mutation of tyrosyl‐DNA phosphodiesterase 1 (TDP1), a gene that resolves DNA replication stress because of abortive topoisomerase reactions and associated strand breaks,^72^ causes spinocerebellar ataxia with axonal neuropathy type 1 (SCAN1), a slowly progressive cerebellar ataxia and sensorimotor polyneuropathy.^73^

Aside from AT, the remaining described disorders of DNA repair and genomic stability are still predominantly in the early pre‐clinical stages of therapy development. Viral‐mediated gene therapy efforts in particular have faced challenges as viral gene overexpression is poorly compatible with the heavily regulated nature of these protein factors within cells.^74^

## Disorders of Enzyme/Substrate Modification

### Cerebrotendinous Xanthomatosis

Cerebrotendinous xanthomatosis (CTX) (Table 2) is caused by mutations in the *CYP27A1* gene, which encodes for sterol 27‐hydroxylase.^75^ It is involved in cholesterol and bile acid synthesis and in its absence, there is a decrease in bile acids such as chenodeoxycholic acid (CDCA) and an increase in pathogenic cholesterol intermediates such as cholestanol. These intermediates can accumulate in the brain causing metabolic derangement and apoptosis. The mainstay of treatment is CDCA supplementation, which improves bile acid composition, lowers serum cholestanol, and results in long term clinical improvements. Early treatment is better with a critical window in the mid 20s^76^ and efforts for newborn screening are underway.^77^ In a consensus statement by modified DELPHI criteria, there was unanimous consensus for the use of CDCA and 78% consensus for the use of CDCA and a HMG‐CoA reductase inhibitor.^78^ Gene therapy using AAV8 with an artificial liver‐specific promoter to drive expression of CYP27A rescued the biochemical abnormalities including bile acids and serum cholestanol in mice lacking *CYP27A1*.^79^ The liver is readily targeted with AAVs and low level of expression is needed for near normalization of the biochemical abnormalities in the mouse. However, immune reaction against the AAV capsid and the exogenous CYP27A1 protein is a major factor limiting clinical translation.^80^

**TABLE 2:** Disorders of Enzyme/Substrate Modification

Disease	Gene(s) implicated	Neurologic symptoms	Current treatment	Under investigation
Cerebrotendinous xanthomatosis	*CYP27A1*	Cerebellar ataxia	Chenodeoxycholic acid (CDCA)^78^	AAV‐mediated gene replacement therapy^79^
		Cognitive dysfunction	HMG‐CoA reductase inhibitor^78^	
		Peripheral neuropathy		
		Pyramidal symptoms		
Ataxia with vitamin E deficiency	*TTPA*	Cerebellar ataxia	High dose vitamin E	
		Dysarthria		
		Diminished deep tendon reflexes		
		Loss of positional and vibration sense		
		Nystagmus		
Adult polyglucosan body disease	*GBE1*	Neurogenic bladder	Symptomatic^81^	AAV‐mediated gene replacement therapy^86^
		Spastic paraplegia		AAV‐mediated gene editing targeting *Gys1* ^84^
		Cerebellar ataxia		AAV‐mediated AmiRNA targeting *Gys1* ^83^
		Sensorimotor axonal peripheral neuropathy		Guaicol to reduce GYS1^85^
		Autonomic dysfunction		144DG11 to enhance autolysosomal degradation of glycogen^89^
		Cognitive difficulties		
Riboflavin transporter deficiency	*SLC52A2*	Sensorineural hearing loss	High dose riboflavin^95^	Esterified riboflavin^96^
	*SLC52A3*	Limb muscle weakness		
		Facial nerve/bulbar palsy		
		Ataxia		
		Respiratory muscle weakness		
		Optic atrophy and visual impairment		

### Adult Polyglucosan Body Disease

Adult polyglucosan body disease (APBD) (Table 2) is caused by mutations in the *GBE1* gene encoding the glycogen branching enzyme protein (GBE1). It is a glycogen storage disorder (GSD) characterized by the accumulation of insoluble glycogen known as polyglucosan bodies.^81^ Reducing glycogen as a substrate is a common therapeutic strategy for GSDs and has shown efficacy in pre‐clinical models via different modalities such as small interfering RNA (siRNA), small molecules, AAV encoding artificial microRNA (AmiRNA), and gene editing in APBD and related GSDs.^82^, ^83^, ^84^, ^85^ Intravenous AAV‐mediated gene replacement therapy improved pathology in the muscle and liver, but not the brain in a mouse model because of poor transduction across the blood–brain barrier.^86^ The most common GBE1 mutation (p.Y329S), common in the Ashkenazi population, yields an unstable protein and a molecular chaperone has been shown to stabilize the protein in vitro.^87^ Enzyme replacement (eg, alglucosidase alfa, used in Pompe disease) failed to reduce polyglucosan bodies in the brain in another disorder (Lafora disease) and is, therefore, unlikely to be effective in APBD.^88^ An autophagy inducer discovered through a small molecule screen has shown efficacy in the mouse model and now has a FDA Orphan drug designation.^89^ Last, anaplerotic dietary therapy using Triheptanoin was found to be promising in a case series of patients with APBD,^90^ however, a double‐blind, placebo‐controlled crossover trial with Triheptanoin did not show efficacy over a 6‐month treatment period.^91^

### Ataxia with Vitamin E Deficiency

Ataxia with vitamin E deficiency (AVED) (Table 2) is caused by mutations in the *TTPA* gene encoding the α‐tocopherol transfer protein (⍺‐TTP).^92^ Despite normal intestinal absorption, serum vitamin E is reduced because of the lack of cytoplasmic transport via ⍺‐TTP into the serum lipoprotein. Vitamin E acts as a potent antioxidant, and its absence can result in oxidative damage, especially in the cerebellar Purkinje cells.^93^ Treatment with up to 50‐ to 100‐fold of the recommended daily amount of vitamin E results in appropriate serum levels. When treated early, it leads to long term stabilization of clinical symptoms, but no improvement of the cerebellar deficits.^94^

### Riboflavin Transporter Deficiency

Riboflavin transporter deficiency (RTD) (Table 2), previously known as Brown‐Vialetto‐Van Laere syndrome, is caused by mutations in the *SLC52A2* (RTD2) and *SLC52A3* (RTD3) genes, which encode the riboflavin transporter. It is characterized by impaired riboflavin absorption across the luminal side of the intestinal cell (RTD3) and into the cell (RTD2). Supplementation of high dose riboflavin improves symptoms for 80.9% of the patients and stabilizes the disease in the rest. It is especially helpful for the motor symptoms, improving the gross motor function classification system by at least 1 level in 93.3% of the patients.^95^ Serum acylcarnitine profiles, which can be abnormal in the disease, are normalized after treatment.^95^ Although riboflavin showed no improvements in the drosophila models of RTD, esterified riboflavin, which is expected to be a transporter‐independent riboflavin pro‐drug, was able to normalize the phenotype when administered from birth.^96^ Unfortunately, the therapeutic index for such a compound may be low as the esterified riboflavin may abnormally deposit intracellularly.

## Disorders of Coenzyme Q10 Deficiency

### COQ8A‐Ataxia

**FIGURE 2:**
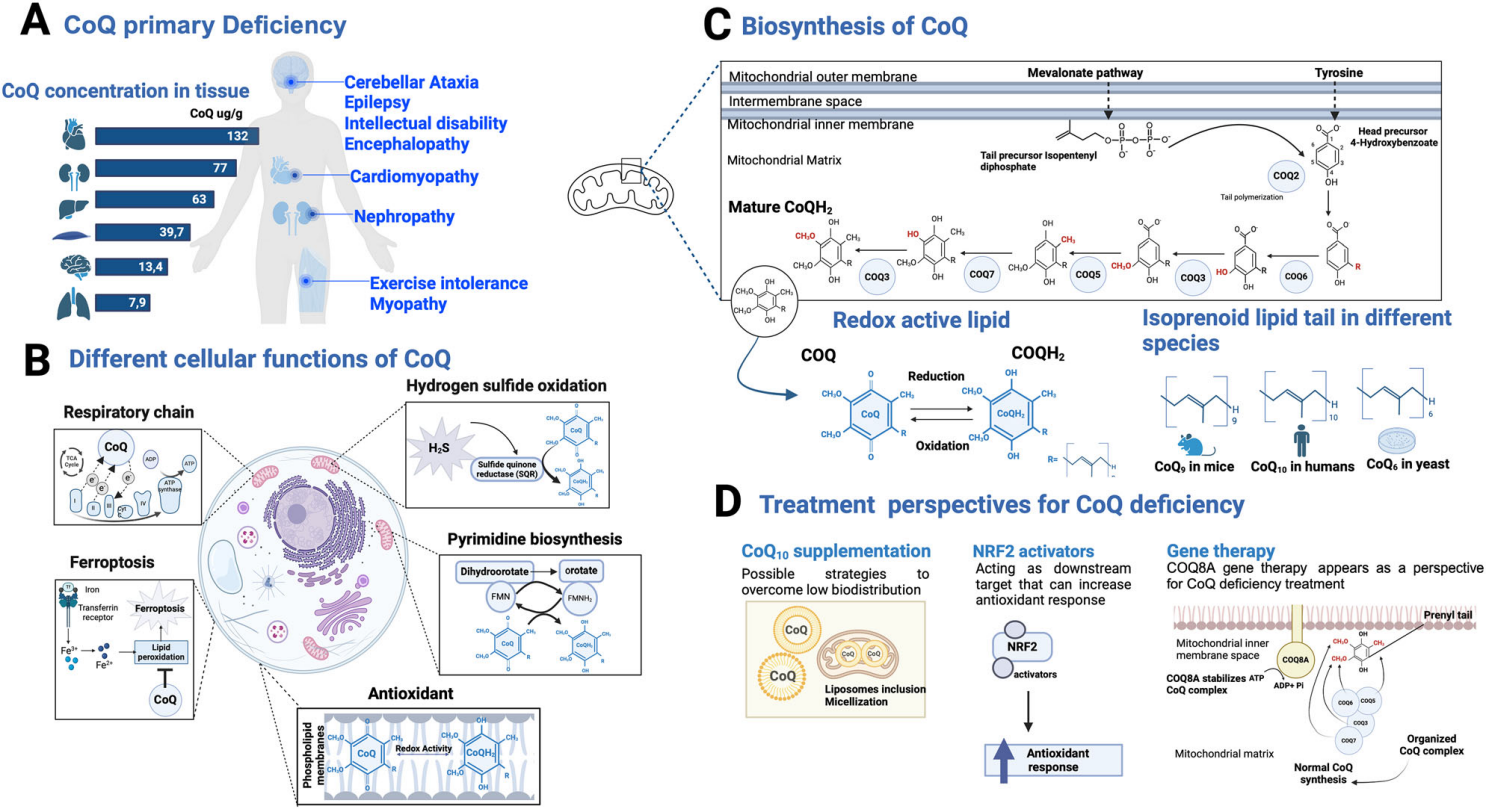
Coenzyme Q (CoQ) biosynthesis, cellular functions, biodistribution, deficiency, and treatment perspectives. (A) CoQ deficiency is a heterogeneous group of rare inherited mitochondrial disorders that reduces CoQ concentration leading to multisystem and diverse clinical features. Despite its devastating consequences in daily life on mobility and communication and reduced life span, no disease‐modifying treatment is available for CoQ deficiency. (B) CoQ functions as an essential cofactor in multiple cellular processes, including mitochondrial oxidative respiration, pyrimidine biosynthesis, sulfide detoxification, ferroptosis, and antioxidation. (C) CoQ is a redox active lipid found in all membranes. Although it is possible to get it from food, most of the CoQ pool that is physically required is synthesized endogenously in the mitochondria. In eukaryotes, CoQ quinone precursor derives from aromatic amino acids such as tyrosine and is imported into mitochondria where it becomes prenylated with precursors generated by the mevalonate pathway. The quinone head group is then maturated through addition of different functional groups, this process occurs within a dynamic complex of COQ proteins (complex Q or CoQ‐synthome) at the inner mitochondrial membrane. Mature CoQ comprises a redox‐active quinone head that can carry electrons and an extremely hydrophobic isoprenoid lipid tail that differs in length depending on the species. (D) CoQ10 supplementation may potentially address CoQ10 deficiency, however, its poor bioavailability is a challenge for treatment efficacy. Various strategies have been explored to increase the biodistribution of CoQ, including mitochondrial targeting moieties, encapsulation in liposomes, nanoparticles and micelles, other treatment perspectives including downstream targets as general antioxidants and NRF2 activators, as well as gene therapy to act upstream of the disease cascade. [Color figure can be viewed at www.annalsofneurology.org]

Primary coenzyme Q10 deficiency constitutes a diverse group of rare inherited mitochondrial disorders characterized by multisystem and diverse clinical features (Fig 2A).^97^ Coenzyme Q (CoQ; ubiquinone) is a redox‐active lipid present in all membranes, serving as an essential cofactor in various cellular processes (Fig 2B).^98^ COQ8A‐ataxia (also known as ADCK3 or autosomal recessive cerebellar ataxia type 2 [ARCA2]) is the most common autosomal recessive cerebellar ataxia related to primary CoQ10 deficiency.^99^, ^100^, ^101^ COQ8A is thought to play a role in maintaining complex Q integrity and regulating CoQ biosynthesis.^102^ The current model is that COQ8A leverages its ATPase activity to support the formation of complex Q, potentially coupled with the extraction of lipid intermediates from the inner mitochondrial membrane to allow chemical modification of the head group by the catalytic CoQ proteins.^103^

Studies using patient fibroblasts and muscle biopsies have revealed that COQ8A deficiency can lead to decreased CoQ10 levels, resulting in inefficient oxidative phosphorylation.^99^ Mouse models with constitutive *COQ8A* deletion have shown complex Q destabilization in all tissues and variable reductions in CoQ levels in different tissues.^102^ These models have also demonstrated that COQ8A deficiency in Purkinje neurons leads to alterations in mitochondrial and calcium homeostasis,^104^ ultimately causing alteration in their pacemaking activity and their specific degeneration.^102^ Furthermore, primary cultures of COQ8A‐deficient Purkinje neurons treated with exogenous CoQ10 showed reversal of morphological defects and restoration of mitochondrial and calcium homeostasis, indicating the potential effectiveness of CoQ treatment in COQ8A‐ataxia.^104^

Although CoQ10 supplementation may potentially address diseases related to primary defects in the CoQ10 biosynthetic pathway, in COQ8A‐ataxia, the clinical response to CoQ supplementation has been limited and highly variable. In the largest multicenter report, only 43% of patients responded to oral CoQ treatment.^101^ Response to therapy can depend on multiple factors, including genetic background,^105^ quality of the CoQ formulation, and timing, dosage, and duration of treatment. Oral CoQ10 has poor bioavailability, approximately 5%, and the degree to which it penetrates into the human brain is unclear,^106^ so there is an urgent need to develop novel tools to improve cellular uptake of CoQ (Fig 2C). Although analogs with shorter isoprenoid chains, like idebenone, show promise for improved bioavailability, idebenone in COQ8A‐ataxia has yielded disappointing results, exacerbating the phenotype in some patients. Numerous strategies have been explored to enhance biodistribution of CoQ (Fig 2D, see Supplemental Material), including mitochondrial targeting moieties and lipid dispersions. An intravenous ubidecarenone‐lipid conjugate nanodispersion of CoQ10 (BPM31510), achieving up to 200‐fold physiological plasma levels,^107^ is now in clinical trials for various diseases, including COQ8A‐ataxia.

## Disorders of Mitochondrial DNA Maintenance

Cerebellar ataxia is one of the most frequent phenotypes of mitochondrial disorders, indicating increased vulnerability of Purkinje cells and spinocerebellar tracts to mitochondrial dysfunction. Loss of Purkinje neurons is found neuropathologically in human postmortem tissues and several mouse models of mitochondrial diseases.^108^ The mitochondrial DNA (mtDNA) maintenance disorders are because of nuclear gene defects affecting proteins needed for mtDNA replication and maintenance leading to quantitative reduction in mtDNA copy number (mtDNA depletion), qualitative alterations (multiple deletions of mtDNA), or both. Accordingly, these disorders are also designated mtDNA depletion and multiple deletions syndromes (MDDS), the most prominent being associated with defects in the gene for mtDNA polymerase subunit gamma 1 (*POLG*).

*POLG*‐associated disorders comprise a wide spectrum of phenotypes with cerebellar and/or sensory ataxia particularly prominent in adolescent‐ to adult‐onset manifestations^109^ with disease‐modifying treatment approaches currently being pursued. Elamipretide is a small synthetic tetrapeptide that can readily penetrate mitochondria where it associates with cardiolipin in the inner mitochondrial membrane. It can partly restore the curvature of this membrane as well as respiratory chain supercomplex formation, thereby enhancing ATP synthesis and reducing reactive oxygen species production. Although a phase 3 study of elamipretide in 218 patients with primary mitochondrial myopathy did not meet its primary endpoints, a post hoc analysis showed that the subgroup of patients with nuclear gene defects (including *POLG*) performed significantly better in the 6‐minute walk test as compared to placebo.^110^ An additional 48‐week randomized, double‐blind, parallel‐group, placebo‐controlled trial of elamipretide in this cohort was completed in September 2024 (NCT05162768) and results are pending. Other therapies include in vitro supplementation of deoxyribonucleotide triphosphates or their precursors, assuming improved substrate availability will help sustain mtDNA replication. In neural stem cells derived from two *POLG* patients, deoxyribonucleoside supplementation led to reduction of ethidium bromide (EtBR)‐induced mtDNA depletion and a marked increase of mtDNA repopulation rates (4‐fold and 5‐fold vs baseline, respectively) after EtBr removal.^111^ As a similar therapy is already being tested in clinical trials for another mitochondrial disease, thymidine kinase 2 deficiency,^112^ this may considerably shorten the development for *POLG*‐associated disorders and other MDDS.

## Disorders of Intracellular Calcium Signaling

**FIGURE 3:**
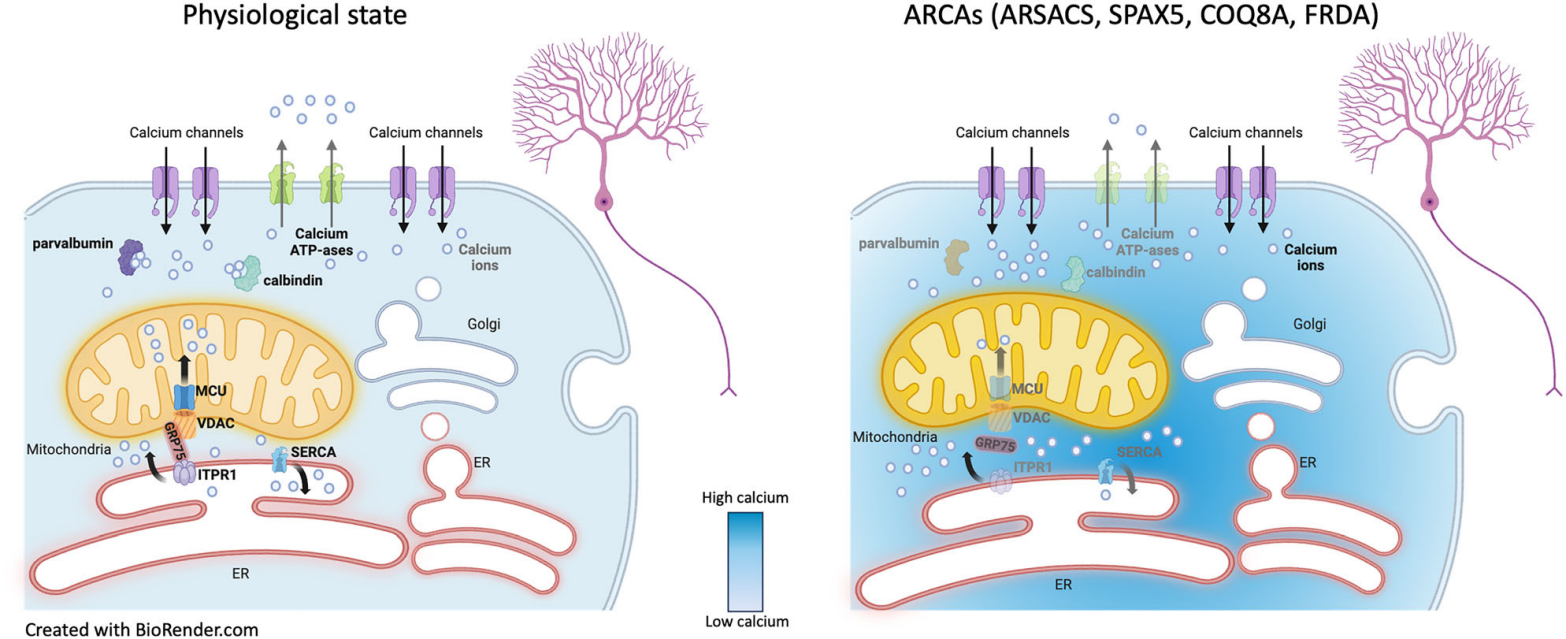
Alteration of calcium handling in autosomal recessive cerebellar ataxias (ARCAs). In ARCAs, alteration of mitochondrial transport (biallelic AFG3L2 [SPAX5] and ARSACS), of endoplasmic reticulum (ER) transport (ARSACS), of mitochondrial calcium buffering (biallelic AFG3L2 [SPAX5], COQ8A, Friedreich ataxia [FRDA]), alterations of mitochondria‐associated membranes (COQ8A, FRDA), and/or deregulation of calcium binding proteins and calcium ATP‐ases at both the plasma membrane and ER membrane (ARSACS) lead to pathological elevations in cytosolic calcium concentrations. [Color figure can be viewed at www.annalsofneurology.org]

A number of molecular components are involved in controlling calcium signaling in Purkinje neurons (PNs), including plasma membrane ion channels, G‐protein coupled receptors, calcium binding proteins, and calcium ATP‐ases at both the plasma membrane and endoplasmic reticulum (ER) membrane.^113^ In the biallelic *AFG3L2* mouse model (*Afg3l2* ^−/−^, autosomal recessive spastic ataxia type 5 [SPAX]), calpain‐mediated proteolysis of αII‐spectrin in the cerebellum^114^ provided evidence of calcium‐dependent neurodegeneration as calpains are activated by elevated cytosolic calcium (Fig 3).^115^ In primary *Afg3l2* ^−/−^ PNs, the cytosolic calcium‐evoked responses were strikingly higher versus controls, indicating inefficient buffering and shaping of calcium spikes. This phenotype was caused by the negative synergy of impaired mitochondrial calcium buffering and inefficient transport of swollen mitochondria to distal dendrites, depriving these sites of ATP needed for active calcium clearance.^114^

In autosomal‐recessive spastic ataxia of Charlevoix Saguenay (ARSACS, because of biallelic mutations in *SACS*), abnormally elevated calcium‐rises in primary *Sacs* ^−/−^ PNs were described as the result of defective mitochondria and ER transport to the dendrites, because of intermediate filament cytoskeleton alterations (see Fig 3).^116^ Deregulated calcium homeostasis in the cerebellum of the *Sacs* ^−/−^ mouse was supported by increased auto‐phosphorylation of CaMKIIβ (Ca^2+^‐calmodulin‐dependent protein kinase type II subunit β, which is calcium dependent)^117^ and reduced levels of key calcium‐buffering proteins (eg, calbindin), calcium ATP‐ases, and inositol 1,4,5‐trisphosphate receptor type 1 (ITPR1), a ligand‐gated ion channel that releases calcium from intracellular stores.^116^ Interestingly, altered ITPR1 levels or activity have been also previously observed in several SCAs^118^ highlighting a key role of ITPR1‐mediated calcium signaling in PN physiology. In the ARSACS mouse model, the administration of ceftriaxone, a β‐lactam antibiotic able to reduce glutamate concentration at the inter‐synaptic space,^119^ was effective at both pre‐ and post‐symptomatic stages. This treatment ameliorated the motor phenotype of the *Sacs* ^−/−^ mice, delaying PN degeneration by restoring cytosolic calcium homeostasis and attenuating reactive astrogliosis.^116^ Ceftriaxone has also been shown effective in decreasing post‐synaptic calcium levels in PNs and improving the motor phenotype of the *Afg3l2* ^+/−^ mouse (SCA28 model) and a myotonic dystrophy type 1 (DM1) model.^114^, ^120^

In the COQ8A mouse model described above, COQ8A‐depleted PNs showed pathological calcium elevations, impaired buffering calcium waves, reduction of ITPR1 and increased phosphorylation of CaMKII,^104^ as seen in ARSACS. In the *Coq8a* ^−/−^ mouse the mitochondria‐associated membranes (MAMs), important sites for calcium transport and signaling, were dilated and fragmented.^104^ Alterations in MAMs have also been described in FRDA,^121^ where loss of frataxin resulted in weakening of the voltage‐dependent anion channel‐glucose‐regulated protein 75‐ITPR1 tethering bridge that ensures calcium flow between the mitochondria and ER. Accordingly, deregulation of calcium homeostasis and activation of calpains has been described in FRDA neuronal cells,^122^ and calcium deposits are found in the myocardium of FRDA patients.^123^ Defective calcium handling may be a common hub in ARCAs, where ceftriaxone and other drugs modulating calcium may represent a shared therapeutic option.

## Disorders of the Lysosome

A group of rare monogenic recessive disorders that can present with cerebellar ataxia are the lysosomal storage diseases (LSDs) (Table 3).^124^ Disease‐specific mutations in genes encoding various enzymes result in lysosomal dysfunction and cause macromolecule accumulation in late endocytic compartments leading to cell dysfunction and ultimately death.^124^ The macromolecules stored in LSDs are biochemically diverse and the nature of the stored metabolites is often used as a classification system.^124^ Despite the rarity of LSDs, progress has been made in translating therapies to the clinic, with approvals for some diseases.^124^ For example, multiple enzyme replacement therapies (ERT) have been approved along with small molecule drugs.^124^ ERT can be a major disease modifying therapy (eg, for type 1 Gaucher disease), but these biologics do not cross the blood–brain barrier so are not used to manage LSDs with CNS manifestations. One exception is ERT for CLN2 where ERT is delivered directly to the CNS.^124^ Substrate reduction therapy is an alternative therapeutic approach to ERT and uses small molecule drugs to reduce the biosynthesis of the stored substrate. To date, this is confined to inhibitors of glycosphingolipid biosynthesis with drugs approved for type 1 Gaucher disease and Niemann‐Pick disease type C (NPC).^124^ Many gene therapies are currently in clinical trials for LSDs (Table 3) and base editing approaches are also under pre‐clinical evaluation (ie, NPC).^125^

**TABLE 3:** Lysosomal Storage Diseases and Disorders of Lysosome‐Related Organelles That Present with Cerebellar Ataxia

Lysosomal disorders	Gene name	Current treatment options	Treatments under investigation
GM1 gangliosidosis	*GLB1*	Symptomatic management; off‐label use of substrate inhibitors or neuroprotective agents (eg, ALL)	Gene therapy
GM2 gangliosidosis (Tay‐Sachs)	*HEXA*	Symptomatic management; off‐label use of substrate inhibitors or neuroprotective agents (eg, ALL)	Gene therapy
GM2 gangliosidosis (Sandhoff)	*HEXB*	Symptomatic management; off‐label use of substrate inhibitors or neuroprotective agents (eg, ALL)	Gene therapy
Niemann‐Pick disease type C	*NPC1*	Substrate reduction approved: miglustat ALL (marketing approval pending)	Gene therapy Cyclodextrin
Sialidosis	*NEU1*	Symptomatic management	n/a
Galactosialidosis	*CTSA*	Symptomatic management	n/a
CLN1	*PPT1*	Symptomatic management	n/a
CLN2	*TPP1*	Intraventricular ERT with cerliponase alfa Symptomatic management	Gene therapy: intracisternal AAV9^158^ Intraparenchymal AAVrh10^159^ Eye: subretinal (AAV9) (NCT05791864) Intravitreal ERT (NCT05152914)
CLN3	*CLN3*	Symptomatic management	Gene therapy (NCT03770572) Miglustat (NCT05174039) ASOs
CLN5	*CLN5*	Symptomatic management	Gene therapy (NCT05228145)
CLN6	*CLN6*	Symptomatic management	Gene therapy (NCT04273243)
CLN7	*MFSD8*	Symptomatic management	Gene therapy (NCT04737460) Individualized ASOs^160^
CLN8	*CLN8*	Symptomatic management	n/a
CLN9	*CLN9*	Symptomatic management	n/a
CLN10	*CTSD*	Symptomatic management	n/a
CLN11	*GRN*	Symptomatic management	Gene therapy: NCT004408625 NCT04747431 NCT06064890 Ab therapy: NCT04374136
Griscelli syndrome	*MYO5A*	Symptomatic management; stem cell transplant	Hematopoietic stem cell transplant; NCT01652092
Chediak‐Higashi	*LYST*	Symptomatic management; stem cell transplant	Hematopoietic stem cell transplant; NCT01652092 NCT01821781
Multiple sulfatase deficiency	*SUMF1*	Symptomatic management	n/a

ALL = Acetyl‐L‐leucine; ASO = antisense oligonucleotides (s); CLN = ceroid lipofuscinosis, neuronal; ERT = enzyme replacement therapy; n/a = none available.

One drug that has more recently emerged as a potential therapy for LSDs is acetyl‐L‐leucine (ALL). It was discovered that acetyl‐DL‐leucine (ADLL) was beneficial for improving the symptoms of cerebellar ataxia^126^ and later in individuals with LSDs including NPC and the GM2 gangliosidoses (Tay‐Sachs and Sandhoff) (Table 3).^127^ Acetyl‐L‐leucine (ALL) has been evaluated in phase 2b clinical trials in NPC (NCT03759639) and the GM2 gangliosidoses (NCT03759665). In both diseases, ALL showed significant benefit.^128^, ^129^ ALL was investigated in a phase 3 placebo controlled cross‐over study in 60 NPC patients (NCT05163288).^130^ Efficacy was demonstrated with statistically significant improvement in the Scale for the Assessment and Rating of Ataxia (SARA) and multiple secondary endpoints.^131^ Although further independent confirmation is still warranted, ALL has been approved by the FDA for the treatment of the neurological symptoms of NPC in September 2024 and a phase III trial has also been initiated for its use in AT^132^ as of November 2024 (NCT06673056).

## Disorders of Protein Quality Control

**FIGURE 4:**
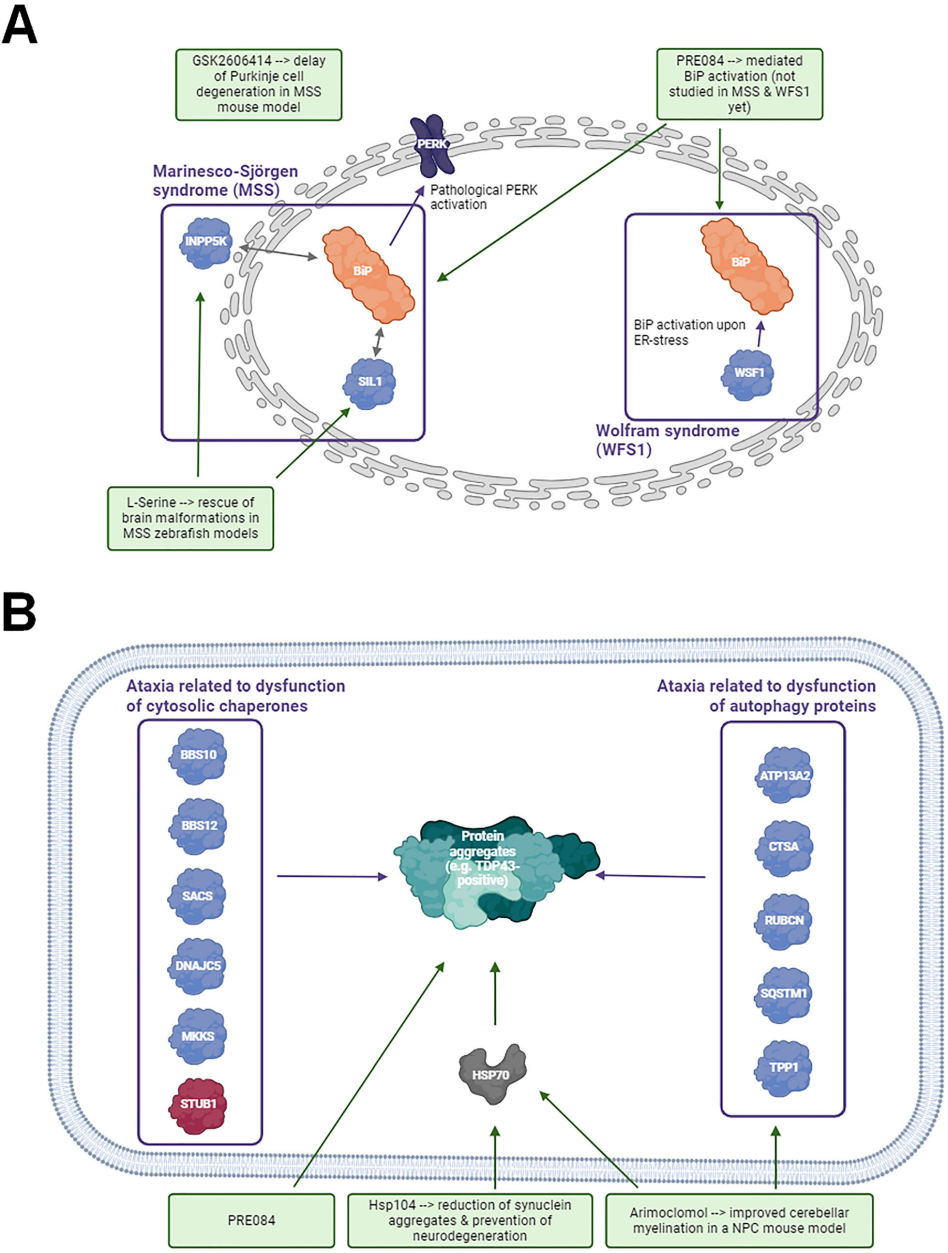
Protein quality control in autosomal recessive cerebellar ataxias (ARCAs). Schematic representation of ataxia‐causative proteins involved in endoplasmic reticulum (ER)‐related protein folding (A) or cytosolic‐related protein‐folding/processing (left) and protein clearance (right) (B). Disease groups are indicated by purple‐framed boxes and medications/active substances (some with pre‐clinical testing results) with therapeutic potential are shown in green boxes. Dark red‐labelling of STUB1 is based on the potential risk of therapeutic activation of a missense‐mutant chaperone with toxic gain‐of‐function. [Color figure can be viewed at www.annalsofneurology.org]

A variety of ataxia genes play crucial roles in protein quality control including folding, transport across compartments, and modulation of degradation (Fig 4; Tables 4, 5, 6). This underlines the need for suitable drugs targeting various perturbed cascades of protein quality control. Different forms of ataxia caused by perturbed ER‐function (including protein folding) might benefit from enhanced folding capacity. For instance, loss of SIL1 (associated with Marinesco‐Sjogren syndrome), a co‐factor of BiP (a major ER‐resident chaperone) is related to profound dysregulation of ER‐function and protein aggregation in PNs within the vestibulocerebellum leading to cellular death.^133^ Moreover, ataxia was described in a patient with bi‐allelic variants in *INPP5K*, encoding another BiP binding protein.^134^ Both disease conditions are biochemically associated with dysregulation of PHGDH, a protein involved in L‐serine metabolism and L‐serine supplementation yielded amelioration of brain‐malformations in zebrafish models of these diseases, consequently introducing L‐serine treatment as a promising treatment concept.^134^ Given that ER‐stress on loss of functional SIL1 is associated with activation of PERK (a modulator of ER‐stress) in degenerating neurons and PERK‐inhibition has protective effects on protein‐misfolding in models of neurodegenerative disease, modulation of PERK‐activation by GSK2606414 (a selective PERK‐inhibitor) was tested.^135^ Chronic treatment of presymptomatic *Sil1*‐mutant mice resulted in delay of Purkinje cell degeneration and onset of motor deficits. Biochemically this was accompanied by cerebellar increase of ORP150/GRP170, an alternative BiP co‐factor,^135^ that rescues neurodegeneration in *Sil1*‐mutant mice.^136^ DNAJC3 /p58(IPK) is another BiP co‐factor linked to ataxia^137^ and loss of DNAJC3/p58(IPK) improves ER stress and neurodegeneration in *Sil1*‐mutant mice.^136^ Interestingly, ER‐stress on loss of Wolframin—mutated in autosomal‐recessive WFS1/Wolfram Syndrome‐associated ataxia—is defined by BiP dysregulation.^138^ BiP‐function can therapeutically be addressed by PRE084, a sigma receptor agonist that increases BiP abundance (see Fig 4).^139^ However, as this approach relies on availability of functional co‐chaperones facilitating proper BiP functions, SIL1 deficiency might be addressable by PRE084 as its role as an ATP‐ADP exchange factor can (partially) be compensated by ORP150/GRP170, whereas DNAJC3/p58(IPK) instead facilitates ATP hydrolysis toward BiP function. In addition, PRE084 confers protection against protein aggregate toxicity (as demonstrated based on TDP‐43 aggregation).^139^ Hence, one might assume that ataxia‐related protein‐aggregation pathologies caused by the presence of pathological variants in ataxia genes encoding cytoplasmic chaperones (*BBS10*, *BBS12*, *DNAJC5*, *MKKS*, and *SACS*) or protein‐clearance factors (such as *IRF2PBL*, *LRSAM1*, *PEX2*, *PEX10*, *RNF216*, *STUB1*, *UBA5*, and *UCHL1*) might benefit from PRE084‐treatment resulting in reduced build‐up of (toxic) protein‐aggregates (see Supplemental Material).

**TABLE 4:** Disorders of Protein Quality Control: Proteins of the Lysosome and the Peroxisome

Gene	Protein	Localization	Function	Disease
ATP13A2/PARK9	Polyamine‐transporting ATPase 13A2	Autophagosome	Lysosome fusion and autophagy	Kufor‐Rakeb syndrome (KRS)(MIM: 606693)
*CTSA*	Lysosomal protective protein	Lysosome	Lysosomal protective protein	Galactosialidosis (GSL)(MIM: 256540)
*NPC1*	NPC intracellular cholesterol transporter 1	Lysosome	Egress of cholesterol from the endosomal/lysosomal compartment	Niemann‐Pick disease, type C1 (MIM: 257220)
*PEX2*	Peroxisome biogenesis factor 2	Peroxisome	Peroxisome organization	Peroxisome biogenesis disorder (Zellweger) (MIM: 614867 and 614866)
*PEX10*	Peroxisome biogenesis factor 10	Peroxisome	Peroxisome organization	Peroxisome biogenesis disorder (Zellweger) (MIM: 614870 and 614871)
*RUBCN*	Run domain Beclin‐1‐interacting and cysteine‐rich domain‐containing protein	Lysosome	Impairs autophagosome maturation process	Spinocerebellar ataxia, autosomal recessive, 15 (SCAR15) (MIM: 615705)
*TPP1*	Tripeptidyl‐peptidase 1	Lysosome	Generates tripeptides from lysosomal proteinase products	Spinocerebellar ataxia, autosomal recessive, 7 (SCAR7) (MIM: 609270)

**TABLE 5:** Disorders of Protein Quality Control: Proteins of the ER and/or Golgi Apparatus

Gene	Protein	Localization	Function	Disease
*DNAJC3*	DnaJ homolog subfamily C member 3	ER	Co‐chaperone that promotes ATP hydrolysis by BiP	Ataxia, combined cerebellar and peripheral, with hearing loss and diabetes mellitus (MIM: 616192)
*INPP5K*	Inositol polyphosphate 5‐phosphatase K	Cytosol and ER	Inositol phosphate signaling, cytoskeleton, and insulin signaling; binding partner of BiP (GRP78, HSPA5)	Muscular dystrophy, congenital, with cataracts and intellectual disability (MIM: 617404)
*ITM2B*	Integral membrane protein 2B	Cell membrane, endosomes, Golgi apparatus	Inhibitor of amyloid‐β peptide aggregation	Cerebral amyloid angiopathy, ITM2B‐related 2 (CAA‐ITM2B2) (MIM: 117300)
*SCYL1*	N‐terminal kinase‐like protein	ER and Golgi apparatus	Protein traffic at the interface between the Golgi apparatus and the ER	Spinocerebellar ataxia, autosomal recessive, 21 (SCAR21) (MIM: 616719)
*SIL1*	Nucleotide exchange factor SIL1	ER	ER‐resident nucleotide exchange factor for BiP	Marinesco‐Sjögren syndrome (MSS) (MIM: 248800)
*TRAPPC11*	Trafficking protein particle complex subunit 11	ER and Golgi apparatus	Involved in ER to Golgi apparatus trafficking at a very early stage	Muscular dystrophy, limb‐girdle, autosomal recessive 18 (MIM: 615356)
*UBA5*	Ubiquitin‐like modifier‐activating enzyme 5	Cytoplasm, nucleus, ER and Golgi apparatus	Specifically catalyzes the first step in ufmylation	Spinocerebellar ataxia, autosomal recessive, 24 (SCAR24) (MIM: 617133)
*UCHL1*	Ubiquitin carboxyl‐terminal hydrolase isozyme L1	Cytoplasm and ER	Ubiquitin carboxyl‐terminal hydrolase isozyme (deubiquitinase)	Spastic paraplegia 79A, autosomal dominant, with ataxia (SPG79A) (MIM: 620221)
*WFS1*	Wolframin	ER	Negatively regulates the ER stress response and positively regulates the stability of V‐ATPase subunits ATP6V1A and ATP1B1	Wolfram syndrome 1 (MIM: 222300)

ER = endoplasmic reticulum.

**TABLE 6:** Disorders of Protein Quality Control: Proteins of the Nucleus, Cytoplasm, Mitochondria, and Extracellular Space

Gene	Protein	Localization	Function	Disease
BBS10	Bardet‐Biedl syndrome 10 protein	Basal body of the primary cilium	Protein folding	Bardet‐Biedl syndrome (MIM: 615987)
*BBS12*	Bardet‐Biedl syndrome 12 protein	Basal body of the primary cilium	Chaperone	Bardet‐Biedl syndrome (MIM: 615989)
*CLPB*	Mitochondrial disaggregase	Mitochondria	Maintain solubility of key mitochondrial proteins	3‐methylglutaconic aciduria 7A and 7B (MGCA7A, 7B) (MIM: 619835, 616,271)
*DNAJC19*	Mitochondrial import inner membrane translocase subunit TIM14	Mitochondria	Mitochondrial co‐chaperone	3‐methylglutaconic aciduria 5 (MGCA5) (MIM: 610198) and dilated cardiomyopathy with ataxia syndrome
*DNAJC5*	DnaJ homolog subfamily C member 5	Cytoplasm	Chaperone	Ceroid lipofuscinosis, neuronal, 4B (Kufs type), autosomal dominant (CLN4B) (MIM: 162350)
*IRF2BPL*	Probable E3 ubiquitin‐protein ligase IRF2BPL	Nucleus	Proteasome‐mediated ubiquitin‐dependent degradation of target proteins	Neurodevelopmental disorder with regression, abnormal movements, loss of speech, and seizures (NEDAMSS) (MIM: 618088)
*LRSAM1*	E3 ubiquitin‐protein ligase LRSAM1	Cytoplasm	E3 ubiquitin‐protein ligase that mediates monoubiquitination	Ataxic form of Charcot–Marie‐Tooth type 2 (MIM: 614436)
*MKKS*	Molecular chaperone MKKS	Cytoplasm and cytoskeleton	Chaperone that assists protein folding on ATP hydrolysis	McKusick‐Kaufman syndrome and Bardet‐Biedl syndrome (MIM: 236700, 605,231)
*RNF216*	E3 ubiquitin‐protein ligase RNF216	Cytoplasm	Ubiquitination	Gordon Holmes syndrome (GDHS) (MIM: 212840)
*SACS*	Sacsin/ DnaJ homolog subfamily C member 29	Cytoplasm	Co‐chaperone that acts as a regulator of the Hsp70 chaperone machinery	Spastic ataxia Charlevoix‐Saguenay type (SACS) (MIM: 270550)
*SQSTM1*	Sequestosome‐1	Nucleus, cytoplasm, autophagosome	Autophagy receptor required for selective macroautophagy (aggrephagy)	Neurodegeneration with ataxia, dystonia and gaze palsy with childhood‐onset (MIM: 617145)
*STUB1*	E3 ubiquitin‐protein ligase CHIP	Cytoplasm and nucleus	E3 ubiquitin‐protein ligase that targets misfolded chaperone substrates toward proteasomal degradation	Spinocerebellar ataxia, autosomal recessive, 16 (SCAR16) Spinocerebellar ataxia, autosomal dominant, 48 (SCA48) (MIM: 615768, 618,093)
*VPS13D*	Intermembrane lipid transfer protein VPS13D	Extracellular	Mitophagy	Spinocerebellar ataxia, autosomal recessive 4 (SCAR4) (MIM: 607317)

## Barriers to Establishing Trial Readiness in ARCAS

Putting these mechanistic treatment approaches and hypotheses into treatment trials requires a battery of different elements that presently face significant barriers.

### Limited Natural History

Registry‐inventoried natural history cohorts of genetically stratified ARCA patients have been built up for ARCAs in general (eg, ARCA Registry),^140^ and for specific ARCAs such as FRDA (European Friedrich's Ataxia Consortium for Translational Studies [EFACTS]),^141^ COQ8A (preparing therapies for autosomal recessive ataxias [PREPARE]), RFC1 (RFC1 Natural History Study, NCT05177809) or SPG7 and ARSACS (PROSPAX; NCT04297891). Longitudinal cohort data have increasingly become available for a larger number of ARCAs, including the most frequent (eg, FRDA, ARSACS, SPG7, and RFC1 disease),^10^, ^17^, ^142^ but also rare ARCAs (eg, POLG, AOA2, and ANO10).^10^, ^142^ Yet they are often of variable quality, with many lacking sufficient rigor, granularity, outcomes beyond limited clinician‐reported metrics, and thorough genotype‐specific disease staging, all of which are required for trial outcome and stratification planning.

### Poor Clinician‐Reported Outcomes

Disease progression captured by clinician‐reported outcomes (such as the SARA) is slow in most ARCAs (eg, increase by 0.6 SARA points/year in ARSACS and SPG7 or 0.8–0.9 SARA points/year in FRDA and RFC1),^10^, ^142^ indicating that treatment trials will require long trial durations and/or large trial populations requiring >200–300 ARCA patients^142^ to capture a treatment response. This problem is intensified by several metric shortcomings of the main clinician‐reported outcome used in the ARCA field—the SARA score—that has been shown to substantially limit its responsiveness,^142^ including substantial intra‐individual variability, reaching almost 20% of the entire scale range on repeated testing within 14 days,^143^ and items with poor responsiveness to change (eg, upper limb).^142^

### Novel Outcome Measures Combined with Innovative Rare Disease Trial Modeling

Despite optimized versions of the SARA (eg, the currently developed SCACOMS^144^ or the functional SARA [fSARA]^145^), or other ataxia clinician‐reported outcomes (like the FARS‐E, now considered by the FDA as a potential clinically meaningful endpoint in FRDA),^146^ the resulting reductions in sample sizes will likely be only gradual, leaving the trial field with still too large of sample sizes, unrealistic for studying rare ARCAs. New clinical outcome assessment strategies that could go beyond clinician‐reported outcomes include gait sensors (eg, digital‐motor outcomes with body‐worn sensors, now being explored in ARSACS,^147^ AT,^148^ and SPG7^149^) or molecular biomarkers (eg, neurofilament light chain, now being explored in AT^150^ and RFC1^48^, ^151^). Although fluid biomarkers are available for some ARCAs that can be useful diagnostically (eg, AFP for AT/AOA2),^1^, ^2^, ^3^, ^21^ fluid biomarkers that track disease progression, severity, and/or response to treatment are lacking for most ARCAS with only a few exceptions (eg, vitamin E for AVED or cholestanol for CTX). Overall, robust trial‐like longitudinal capture of any non‐clinician reported outcomes is still starkly absent in most ARCAs (except for FRDA, ARSACS, SPG7, and RFC1, see consortia above), especially with the multi‐center validation, convergent validation by other outcome modalities, and anchoring in patient‐relevant aspects of health, as requested by the FDA for clinical trials usage.^152^ In addition, developing composite measures using different outcome domains might yield substantially larger reductions in cohort sizes. Combining a clinician‐reported examination scale supplemented with molecular (eg, biomarker) or digital‐motor (eg, gait sensor) outcomes might yield substantially larger reductions in sample sizes. A recent study has examined how adding digital‐motor outcome information might improve the SARA performance, and therefore, reduce the number of subjects needed.^153^

### Earlier Genetic Diagnosis and Enrollment in Treatment Trials

A recent multi‐center study analyzing real‐world data and accessibility of ARCA patients demonstrated that most ARCA patients available for trial recruitment in real‐world settings will already be >10 years into their disease, with a substantial share of patients no longer walking independently—in particular from ARSACS, SYNE1, AOA2, or POLG.^10^ This highlights the need for an earlier and faster genetic diagnosis in ARCA patients, by directly using the most time‐effective sequencing technologies in unexplained ataxia patients such as whole genome sequencing and payers allowing testing that fully cover all genes and mutation types.^154^, ^155^

## Future Directions

With improved genetic diagnosis of virtually all patients with mutations in known ARCA genes, clinical focus now needs to be shifted to developing targeted therapies for these genetically stratified ARCA patients and making them clinical trial‐ready.^156^ We have outlined a set of molecular themes for organization of many common ARCAs, themes that implicate specific pathways for therapeutic treatment that are both achievable and effective. The development of patient registries to enhance cohort building in conjunction with longitudinal outcome validation of more sensitive metrics to capture change in a patient‐meaningful fashion is needed to prepare and design future mechanistic clinical trials. In addition, given the rarity and complexity of these diseases and patient populations, the field must develop novel methodology for innovative therapy approaches, outcome measures, and disease and trial modelling specifically tailored to small sample sizes. This represents a paradigm shift in thinking in neurology, with diagnostics serving as a gateway into the development of clinical trials, which is a more theragnostic view of genetic testing. Although ARCAs had long been considered untreatable, these diseases are now increasingly lending themselves to becoming paradigmatic models for the general development of targeted, molecular precision therapies for rare neurological diseases because of their defined genetic and molecular etiology. Given the rarity of individual ARCAs—most of them contributing less than 2% of all cases^10^—this provides a general blueprint development for innovative therapy approaches, outcome measures, and disease and trial modelling in rare neurological diseases, including tailoring efforts to small sample sizes and even n‐of‐few/n‐of‐1 studies.

## Author Contributions

B.L.F. and M.S. contributed to the conception and design of the manuscript; all authors contributed to the interpretation of studies included in the manuscript; B.L.F., M.S., and all other authors contributed to drafting the text and preparing the figures.

## Potential Conflicts of Interests

F.M.P. is an academic co‐founder of IntraBio, the biopharmaceutical company developing acetyl‐L‐leucine (ALL), discussed in this manuscript. The remaining authors have nothing to report.

## Supporting information


**Data S1.** Supporting Information.

## Data Availability

None.
