# Supplementary material for: Autosomal Recessive Cerebellar Ataxias: Translating Genes to Therapies
Source: Ann Neurol. 2025 Jun 4;98(3):448–70. doi: 10.1002/ana.27271 (PMC12392066; doi:10.1002/ana.27271)
Supplement: Supplementary file 1 — Data S1. Supporting Information. [file ANA-98-448-s001.docx]

**SUPPLEMENTAL MATERIAL**

**GENETIC DIAGNOSIS OF AUTOSOMAL RECESSIVE CERBELLAR ATAXIAS**

Progress in next-generation sequencing (NGS) has led to an explosion of novel ARCA genes and phenotypes in the last decade, with >200 recessive conditions now identified,^1,2^ rendering a NGS-first approach the most promising and cost- and time-effective approach in clinical practice.^1,3^ As NGS ataxia panels were superseded by an exome-first approach in terms of cost- and time-efficiency in the late 2010s, a genome-first approach^4,5^ is now becoming increasingly considered as the most effective diagnostic approach for ARCAs. Short-read genome sequencing further allows detection of non-conventional mutation types in ARCAs, such as deep-intronic mutations, frequent, for example, in ataxia-telangiectasia,^6^ and now becoming an attractive treatment target,^6,7^ that would be missed by exome sequencing. For identifying hard-to-solve ARCAs, in the future, short-read genome sequencing (SR-GS) might be complemented – and likely even be replaced – by long-read genome sequencing (LR-GS). While SR-GS techniques produce reads of up to 600 bases, long-read sequencing techniques generate reads >10 kb, which improves de novo gene assembly, mapping certainty, transcript isoform identification, and detection of structural variants.^8^ This advantage of LR-GS allows resolution of complex and long repeat expansion motifs, frequent in genetic ataxias, which are not resolvable by SR-GS, e.g. the *GAA-FGF14* repeat (SCA27B),^9,10^ and other complex structural mutation motifs, such as a combined repeat expansion nested in a copy number variation, e.g. in *GLS*-ataxia.^11^

**Disorders of DNA Repair and Genomic Stability**

**Ataxia-telangiectasia-Like Disorders**

Two additional DNA repair disorders that mimic the clinical presentation of AT, but typically lack telangiectasias, immunodeficiency, and increased alpha‐fetoprotein, are defined as the AT-Like Disorders, ATLD1 and ATLD2 (Figure 1).^12,13^ ATLD1 is caused by mutation of *MRE11* (MRE11 Homolog, Double Strand Break Repair Nuclease), which plays a key role in the activation of ATM in response to DNA damage.^12,13^ ATLD2 is caused by mutation of *PCNA* (proliferating cell nuclear antigen), a DNA damage protein involved in DNA replication.^12,13^ Targeting of ATM related pathways is also an active area of treatment investigation, and related studies indicate that ATM deficiency makes cells more sensitive to cell cycle checkpoint inhibition via ATR (Ataxia telangiectasia and Rad3-related) suggesting that small molecular inhibitors of this protein could be an effective cancer treatment for AT patients and carriers.^14^

**Disorders of Coenzyme Q10 deficiency**

**Improving Coenzyme Q10 Bioavailability**

CoQ consists of a redox-active quinone head group capable of carrying electrons and an extremely hydrophobic isoprenoid lipid tail, and is synthesized endogenously within mitochondria within a dynamic complex of COQ proteins (complex Q or CoQ-synthome) at the inner mitochondrial membrane, which may act as a metabolon.^15,16^ MitoQ, featuring a mitochondrial targeting moiety, demonstrates potent antioxidant properties and specific accumulation in mitochondria *in vivo*, but performs poorly in supporting mitochondrial O_2_ consumption.^17,18^ Another approach involves encapsulating CoQ in mitochondrial-targeted liposomes like the MITO-Porter, although it still faces challenges related to biophysical delivery barriers.^19,20^ Another method involves micellization of CoQ with the fungicide caspofungin, which enhances water solubility and cellular uptake but needs improvement for blood-brain barrier penetration.^21^ COQ8A gene therapy might be utilized, not only for COQ8A-ataxia, but for all CoQ deficiencies. Studies in yeast suggest that overexpressing Coq8, the yeast analog, can mitigate many Coq deficiencies by stabilizing complex Q.^22^ Modulators of CoQ biosynthesis or specific targeting approaches may ultimately be the most effective pharmacological strategies.^23,24^

**Disorders of Protein Quality Control**

**Approaches to Treatment for Disrupted Protein Quality Control**

Arimoclomol is a hydroxylamine derivative acting as a co-inducer of chaperones, such as heat shock proteins, thus elevating cellular protein folding capacity. Treatment of a Niemann-Pick disease type C (NPC) mouse model with Arimoclomol improved cerebellar myelination. Furthermore, treatment of NPC-patient derived fibroblasts with Arimoclomol attenuated lysosomal storage defects and dysfunction.^25^ Results of a clinical study demonstrated that Arimoclomol provided a significant and clinically meaningful treatment effect in NPC-patients and was well tolerated and, in December 2023, the FDA accepted a New Drug Application for Arimoclomol for the treatment of NPC.^26^ Based on these combined findings, one might speculate that Arimoclomol-treatment also may improve lysosomal and autophagosomal defects in other ataxia-related proteins such as ATP13A2, CTSA, RUBCN, SQSTM1, and TPP1. However, pre-clinical studies are needed to confirm this assumption. Regarding the Arimoclomol-based elevation of chaperone expression, it is important to consider potential adverse effects from promoting expression of missense mutant proteins. For example, STUB1, which has E3 ubiquitin ligase activity and where amino acid substitutions may either cause loss of function or may have a toxic gain of function effect,^27,28^ also acts as part of a chaperone complex linking the chaperone system to the cellular degradation machinery.^29^

Recently, recombinant viral vector technology was applied to examine neuroprotection via expression of exogenous proteins modulating Hsp70 function: Hsp104 is a non-mammalian hybrid chaperone that can disaggregate large protein aggregates and hereby rescue proteins trapped within these pathological structures.^30,31^ Given that lentiviral vector-based Hsp104-expression in a rat model of Parkinson disease resulted in decrease of synuclein inclusions and prevented neurodegeneration,^32^ this approach seems also to be promising for certain subtypes of ataxia defined by the build-up of protein aggregates. Here, first functional studies on patient-derived cells would be a suitable pre-clinical approach enabling rapid insights into the effect of Hsp104-overexpression on protein-aggregate formation and cellular fitness.

**Mitochondrial-Related Ataxias, Protein Quality Control, and Calcium Signaling**

Mitochondria health and integrity depend on correct protein import, folding, and regulated turnover termed mitochondrial protein quality control (MPQC). Genetic variants in proteins involved in MPQC have been linked to ataxia. A paradigmatic example is *SPG7,* encoding the mitochondrial inner membrane m-AAA protease component paraplegin (SPG7), a frequent cause of spastic ataxia,^3,33^ which possesses both ATPase and protease activities and ensures protein quality control by degrading misfolded polypeptides.^34^ AFG3L2, another m-AAA protease is required for SPG7 maturation into its active mature form^35^ and pathogenic variants are linked to both recessive (SPAX5) and dominant (SCA28) forms of ataxia, as previously described. This protein acts as a regulator of Ca2+ in neurons by mediating degradation of SMDT1/EMRE before its assembly with the uniporter complex, thus limiting the availability of SMDT1/EMRE for MCU assembly and promoting efficient assembly of gatekeeper subunits with MCU.^36^ *MICU1* encodes one gatekeeper of the mitochondrial Ca2+ uniporter MCU complex, and biallelic loss-of-function mutations cause a complex, neuromuscular disorder in children associated with moderate ataxia.^37^ In line with the finding of cytoplasmic protein aggregates in *MICU1*-patient derived cells,^37^ functional studies unveiled a MICU1-SGPL1-VPS39 axis that stimulates intracellular organelle interactions and sustains autophagy and mitochondrial homeostasis.^38^ An impact of altered proteostasis upon impaired function of binding partners of MCU is moreover supported by the finding of increased phosphorylation of the α-subunit of the eukaryotic translation initiation factor 2 (eIF2α) in terms of UPR-activation in both skin fibroblasts from AFG3L2 patients and in the cerebellum of Afg3l2-/- mice.^39^ These combined findings in turn demonstrate a profound impact of altered mitochondrial function associated with impaired Ca2+ homeostasis on overall cellular protein folding and clearance capacity and along this line suggest that modulation of protein folding and degradation machineries might reflect an therapeutic option in mitochondrial-related forms of ataxia.

**REFERENCES**

1. Synofzik M, Puccio H, Mochel F, Schols L. Autosomal Recessive Cerebellar Ataxias: Paving the Way toward Targeted Molecular Therapies. *Neuron* 2019; **101**(4): 560-83.

2. Traschutz A, Adarmes-Gomez AD, Anheim M, et al. Autosomal Recessive Cerebellar Ataxias in Europe: Frequency, Onset, and Severity in 677 Patients. *Mov Disord* 2023; **38**(6): 1109-12.

3. Ngo KJ, Rexach JE, Lee H, et al. A diagnostic ceiling for exome sequencing in cerebellar ataxia and related neurological disorders. *Hum Mutat* 2020; **41**(2): 487-501.

4. Lionel AC, Costain G, Monfared N, et al. Improved diagnostic yield compared with targeted gene sequencing panels suggests a role for whole-genome sequencing as a first-tier genetic test. *Genet Med* 2018; **20**(4): 435-43.

5. van der Sanden B, Schobers G, Corominas Galbany J, et al. The performance of genome sequencing as a first-tier test for neurodevelopmental disorders. *Eur J Hum Genet* 2023; **31**(1): 81-8.

6. Kim J, Woo S, de Gusmao CM, et al. A framework for individualized splice-switching oligonucleotide therapy. *Nature* 2023; **619**(7971): 828-36.

7. Synofzik M, van Roon-Mom WMC, Marckmann G, et al. Preparing n-of-1 Antisense Oligonucleotide Treatments for Rare Neurological Diseases in Europe: Genetic, Regulatory, and Ethical Perspectives. *Nucleic Acid Ther* 2021.

8. Amarasinghe SL, Su S, Dong X, Zappia L, Ritchie ME, Gouil Q. Opportunities and challenges in long-read sequencing data analysis. *Genome Biol* 2020; **21**(1): 30.

9. Pellerin D, Danzi MC, Wilke C, et al. Deep Intronic FGF14 GAA Repeat Expansion in Late-Onset Cerebellar Ataxia. *N Engl J Med* 2023; **388**(2): 128-41.

10. Wilke C, Pellerin D, Mengel D, et al. GAA-FGF14 ataxia (SCA27B): phenotypic profile, natural history progression and 4-aminopyridine treatment response. *Brain* 2023.

11. Fazal S, Danzi MC, van Kuilenburg ABP, et al. Repeat expansions nested within tandem CNVs: a unique structural change in GLS exemplifies the diagnostic challenges of non-coding pathogenic variation. *Hum Mol Genet* 2023; **32**(1): 46-54.

12. Raslan IR, de Assis Pereira Matos PCA, Boaratti Ciarlariello V, et al. Beyond Typical Ataxia Telangiectasia: How to Identify the Ataxia Telangiectasia-Like Disorders. *Mov Disord Clin Pract* 2021; **8**(1): 118-25.

13. Veenhuis S, van Os N, Weemaes C, Kamsteeg EJ, Willemsen M. Ataxia-Telangiectasia. In: Adam MP, Feldman J, Mirzaa GM, et al., eds. GeneReviews((R)). Seattle (WA); 2023.

14. Biswas H, Makinwa Y, Zou Y. Novel Cellular Functions of ATR for Therapeutic Targeting: Embryogenesis to Tumorigenesis. *Int J Mol Sci* 2023; **24**(14).

15. Guerra RM, Pagliarini DJ. Coenzyme Q biochemistry and biosynthesis. *Trends Biochem Sci* 2023; **48**(5): 463-76.

16. Staiano C, Garcia-Corzo L, Mantle D, et al. Biosynthesis, Deficiency, and Supplementation of Coenzyme Q. *Antioxidants (Basel)* 2023; **12**(7).

17. Kelso GF, Porteous CM, Coulter CV, et al. Selective targeting of a redox-active ubiquinone to mitochondria within cells: antioxidant and antiapoptotic properties. *J Biol Chem* 2001; **276**(7): 4588-96.

18. Suarez-Rivero JM, Pastor-Maldonado CJ, Povea-Cabello S, et al. Coenzyme Q(10) Analogues: Benefits and Challenges for Therapeutics. *Antioxidants (Basel)* 2021; **10**(2).

19. Hibino M, Yamada Y, Fujishita N, et al. The Use of a Microfluidic Device to Encapsulate a Poorly Water-Soluble Drug CoQ(10) in Lipid Nanoparticles and an Attempt to Regulate Intracellular Trafficking to Reach Mitochondria. *J Pharm Sci* 2019; **108**(8): 2668-76.

20. Yamada Y, Harashima H. MITO-Porter for Mitochondrial Delivery and Mitochondrial Functional Analysis. *Handb Exp Pharmacol* 2017; **240**: 457-72.

21. Wang Y, Hekimi S. Micellization of coenzyme Q by the fungicide caspofungin allows for safe intravenous administration to reach extreme supraphysiological concentrations. *Redox Biol* 2020; **36**: 101680.

22. He CH, Xie LX, Allan CM, Tran UC, Clarke CF. Coenzyme Q supplementation or over-expression of the yeast Coq8 putative kinase stabilizes multi-subunit Coq polypeptide complexes in yeast coq null mutants. *Biochim Biophys Acta* 2014; **1841**(4): 630-44.

23. Murray NH, Asquith CRM, Fang Z, et al. Small-molecule inhibition of the archetypal UbiB protein COQ8. *Nat Chem Biol* 2023; **19**(2): 230-8.

24. Murray NH, Lewis A, Rincon Pabon JP, Gross ML, Henzler-Wildman K, Pagliarini DJ. 2-Propylphenol Allosterically Modulates COQ8A to Enhance ATPase Activity. *ACS Chem Biol* 2022; **17**(8): 2031-8.

25. Gray J, Fernandez-Suarez ME, Falah M, et al. Heat shock protein amplification improves cerebellar myelination in the Npc1(nih) mouse model. *EBioMedicine* 2022; **86**: 104374.

26. Mengel E, Patterson MC, Da Riol RM, et al. Efficacy and safety of arimoclomol in Niemann-Pick disease type C: Results from a double-blind, randomised, placebo-controlled, multinational phase 2/3 trial of a novel treatment. *J Inherit Metab Dis* 2021; **44**(6): 1463-80.

27. Chen DH, Latimer C, Yagi M, et al. Heterozygous STUB1 missense variants cause ataxia, cognitive decline, and STUB1 mislocalization. *Neurol Genet* 2020; **6**(2): 1-13.

28. Mengel D, Traschutz A, Reich S, et al. A de novo STUB1 variant associated with an early adult-onset multisystemic ataxia phenotype. *J Neurol* 2021; **268**(10): 3845-51.

29. Kalia SK, Kalia LV, McLean PJ. Molecular chaperones as rational drug targets for Parkinson's disease therapeutics. *CNS Neurol Disord Drug Targets* 2010; **9**(6): 741-53.

30. Glover JR, Lindquist S. Hsp104, Hsp70, and Hsp40: a novel chaperone system that rescues previously aggregated proteins. *Cell* 1998; **94**(1): 73-82.

31. Mosser DD, Ho S, Glover JR. Saccharomyces cerevisiae Hsp104 enhances the chaperone capacity of human cells and inhibits heat stress-induced proapoptotic signaling. *Biochemistry* 2004; **43**(25): 8107-15.

32. Lo Bianco C, Shorter J, Regulier E, et al. Hsp104 antagonizes alpha-synuclein aggregation and reduces dopaminergic degeneration in a rat model of Parkinson disease. *J Clin Invest* 2008; **118**(9): 3087-97.

33. Fogel BL, Lee H, Deignan JL, et al. Exome sequencing in the clinical diagnosis of sporadic or familial cerebellar ataxia. *JAMA Neurol* 2014; **71**(10): 1237-46.

34. Koppen M, Metodiev MD, Casari G, Rugarli EI, Langer T. Variable and tissue-specific subunit composition of mitochondrial m-AAA protease complexes linked to hereditary spastic paraplegia. *Mol Cell Biol* 2007; **27**(2): 758-67.

35. Magri S, Fracasso V, Plumari M, et al. Concurrent AFG3L2 and SPG7 mutations associated with syndromic parkinsonism and optic atrophy with aberrant OPA1 processing and mitochondrial network fragmentation. *Hum Mutat* 2018; **39**(12): 2060-71.

36. Konig T, Troder SE, Bakka K, et al. The m-AAA Protease Associated with Neurodegeneration Limits MCU Activity in Mitochondria. *Mol Cell* 2016; **64**(1): 148-62.

37. Kohlschmidt N, Elbracht M, Czech A, et al. Molecular pathophysiology of human MICU1 deficiency. *Neuropathol Appl Neurobiol* 2021; **47**(6): 840-55.

38. Jackson J, Wischhof L, Scifo E, et al. SGPL1 stimulates VPS39 recruitment to the mitochondria in MICU1 deficient cells. *Mol Metab* 2022; **61**: 101503.

39. Franchino CA, Brughera M, Baderna V, et al. Sustained OMA1-mediated integrated stress response is beneficial for spastic ataxia type 5. *Brain* 2024; **147**(3): 1043-56.
